# Menstrual product choice and uptake among young women in Zimbabwe: a pilot study

**DOI:** 10.1186/s40814-020-00728-5

**Published:** 2020-11-23

**Authors:** Mandikudza Tembo, Jenny Renju, Helen A. Weiss, Ethel Dauya, Tsitsi Bandason, Chido Dziva-Chikwari, Nicol Redzo, Constancia Mavodza, Tendai Losi, Rashida Ferrand, Suzanna C. Francis

**Affiliations:** 1 MRC Tropical Epidemiology Group, London School of Hygiene and Tropical Medicine, London, UK; 2 Biomedical Research and Training Institute, Harare, Zimbabwe; 3 Department of Epidemiology and Biostatistics, Kilimanjaro Christian Medical University College, Moshi, Tanzania; 4 Clinical Research Department, London School of Hygiene and Tropical Medicine, London, UK; 5 Department of Public Health, Environments and Society, Faculty of Public Health and Policy, London School of Hygiene and Tropical Medicine, London, UK

**Keywords:** Menstrual health, Community-based interventions, Adolescents, Youth

## Abstract

**Background:**

Menstrual health and hygiene (MHH) is a human rights issue; yet, it remains a challenge for many, especially in low- and middle-income countries (LMICs). MHH includes the socio-political, psychosocial, and environmental factors that impact women’s menstrual experiences. High proportions of girls and women in LMICs have inadequate MHH due to limited access to menstrual knowledge, products, and stigma reinforcing harmful myths and taboos. The aim of this pilot was to inform the design of an MHH sub-study and the implementation and scale-up of an MHH intervention incorporated into a community-based cluster-randomized trial of integrated sexual and reproductive health (SRH) services for youth in Zimbabwe. The objectives were to investigate (1) uptake of a novel MHH intervention, (2) menstrual product preference, and (3) the factors that informed uptake and product choice among young women.

**Methods:**

Female participants aged 16–24 years old attending the community-based SRH services between April and July 2019 were offered the MHH intervention, which included either a menstrual cup or reusable pads, analgesia, and MHH education. Descriptive statistics were used to quantitatively assess uptake and product choice. Focus group discussions and in-depth interviews with participants and the intervention team were used to investigate the factors that influenced uptake and product choice.

**Results:**

Of the 1732 eligible participants, 1414 (81.6%) took up the MHH intervention at first visit. Uptake differed by age group with 84.6% of younger women (16–19 years old) compared to 79.0% of older women (20–24 years old) taking up the intervention. There was higher uptake of reusable pads (88.0%) than menstrual cups (12.0%). Qualitative data highlighted that internal factors, such as intervention delivery, influenced uptake. Participants noted the importance of access to free menstrual products, analgesics, and MHH education in a youth-friendly environment. External factors such as sociocultural factors informed product choice. Barriers to cup uptake included fears that the cup would compromise young women’s virginity.

**Conclusions:**

Pilot findings were used to improve the MHH intervention design and implementation as follows: (1) cup ambassadors to improve cup promotion, sensitization, and uptake; (2) use of smaller softer cups; and (3) education for community members including caregivers and partners.

**Trial registration:**

Registry: Clinicaltrials.gov

Registration Number: NCT03719521

Registration Date: 25 October 2018

## Introduction

Menstruation is an issue that impacts many facets of life such as social participation, mental and physical health, education, and employment [1–3]. Globally, women and girls face numerous challenges in managing their menstruation. These challenges arise from cultural taboos, lack of knowledge, limited access to safe and secure water, sanitation, and hygiene (WASH) services, affordable menstrual products, appropriate disposal structures, and pain management [4–6]. In 2016, the Joint Monitoring Program of the World Health Organization (WHO) and UNICEF defined adequate menstrual hygiene management as access to clean absorbents including sufficient washing, drying, storage and wrapping of reusable absorbents; adequate frequency of absorbent change; washing the body with soap and water; adequate disposal facilities; privacy for managing menstruation; and basic understanding of menstruation and how to manage it with dignity and without fear or embarrassment [7, 8]. More recently, this has been expanded to menstrual health and hygiene (MHH) to include the sociocultural and economic factors that inform menstrual management and impact women’s lives [3, 8].

Improving MHH will contribute to achieving the Sustainable Development Goals (SGDs) on gender equality (SDG5), good health (SDG3), education (SDG4), and clean water and sanitation (SDG6) [9]. Yet, most girls and women in low and middle-income countries (LMICs) continue to have inadequate MHH [10]. Current options for menstrual products include disposable pads and tampons and cost-effective and environmentally friendly reusable pads, period pants, and the menstrual cup. However, many women and girls from LMICs use ineffective and unhygienic menstrual materials such as cloth, toilet paper, or old underwear [11]. Discomfort during menstruation due to ineffective menstrual products and pain relief may result in heightened anxiety, lack of confidence, and absenteeism from school, work, sports, and other socially or economically beneficial activities [12, 13]. Moreover, lack of accurate menstrual knowledge and the proliferation of harmful sociocultural norms can lead to myths and taboos that also contribute to the negative menstrual experiences of young women in LMICs [3].

A systematic review in 2016 highlighted the potential for interventions to improve MHH-related outcomes among women and girls but noted a lack of evidence on optimum models for delivery of MHH interventions [10]. Since then, several intervention studies have been conducted in LMICs but vary in quality and focus almost exclusively on girls in school [3]. Little is known about how girls and young women respond to or experience non-school, community-based MHH interventions, and which factors inform intervention or product uptake [14].

**Fig. 1:**
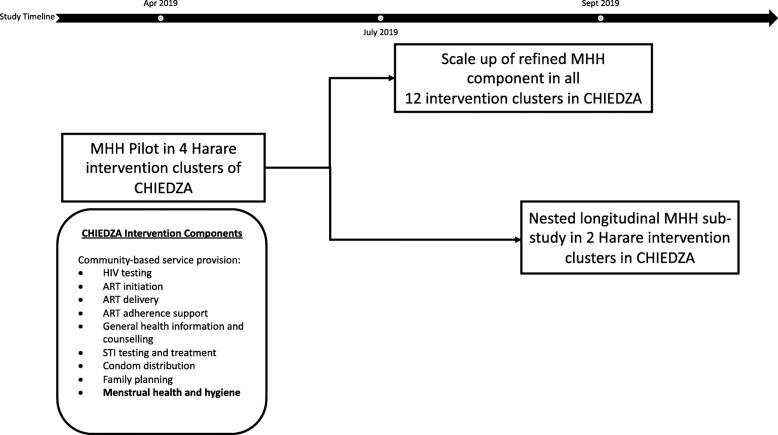
Framework for how the pilot informs the CHIEDZA trial and the nested MHH longitudinal sub-study

The aim of this pilot study was to assess and refine the design and implementation of a comprehensive MHH intervention incorporated into a cluster randomized trial of a package of integrated HIV and sexual and reproductive health (SRH) services for youth delivered in community-based settings across Zimbabwe (the CHIEDZA trial) (registered in clinical trials.gov:NCT03719521). Pilot findings informed two studies: the implementation and scale-up of the MHH intervention within the CHIEDZA trial; and a nested longitudinal MHH sub-study investigating the acceptability, uptake, and effectiveness of an MHH intervention on MHH knowledge, practices, and perceptions among young women aged 16–24 years in Zimbabwe (Fig. 1).

The objectives were to investigate (1) the uptake of a novel comprehensive MHH intervention, (2) menstrual product preference, and (3) the external sociocultural, economic, physical, and environmental factors that informed intervention uptake and menstrual product choice among young women in a community-based SRH programme.

## Methods

### Study setting and participants

This study was nested within the ongoing CHIEDZA trial that seeks to determine the impact of an integrated community-based package of SRH and HIV services for 16–24 year olds on population-level HIV prevalence and other health outcomes. The two-arm trial is conducted in 24 clusters (a geographically demarcated area that includes a community centre and a primary health care clinic) in three provinces in Zimbabwe (Harare, Mashonaland East, and Bulawayo), with eight clusters per province. Each province is stratified 1:1 to either existing, routine health services (control arm), or to receive a package of SRH and HIV services (intervention arm). The intervention arm services are delivered in community centres over a 2-year period. All residents aged 16–24 years in the intervention clusters are eligible to access CHIEDZA services.

In the current paper, we describe an MHH pilot study which included female participants accessing CHIEDZA services at the four intervention clusters in the poor and urban settings of the Harare province.

### The MHH intervention

**Fig. 2:**
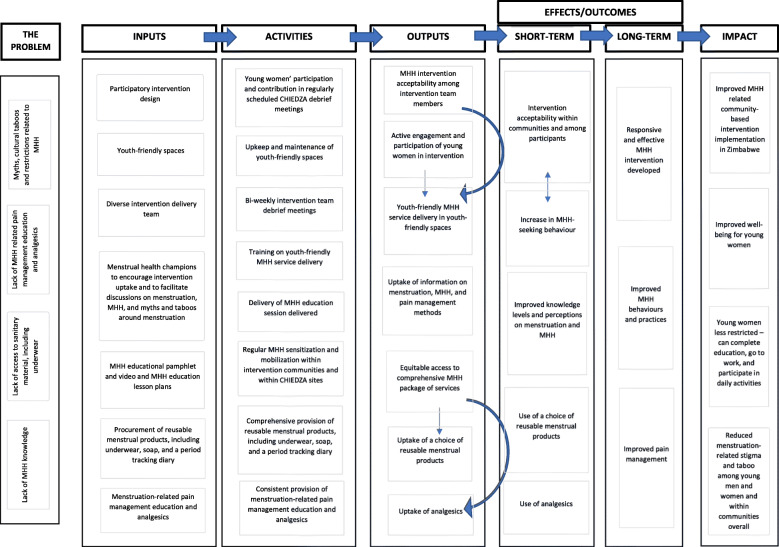
Theory of Change for the MHH Intervention

**Fig. 3:**
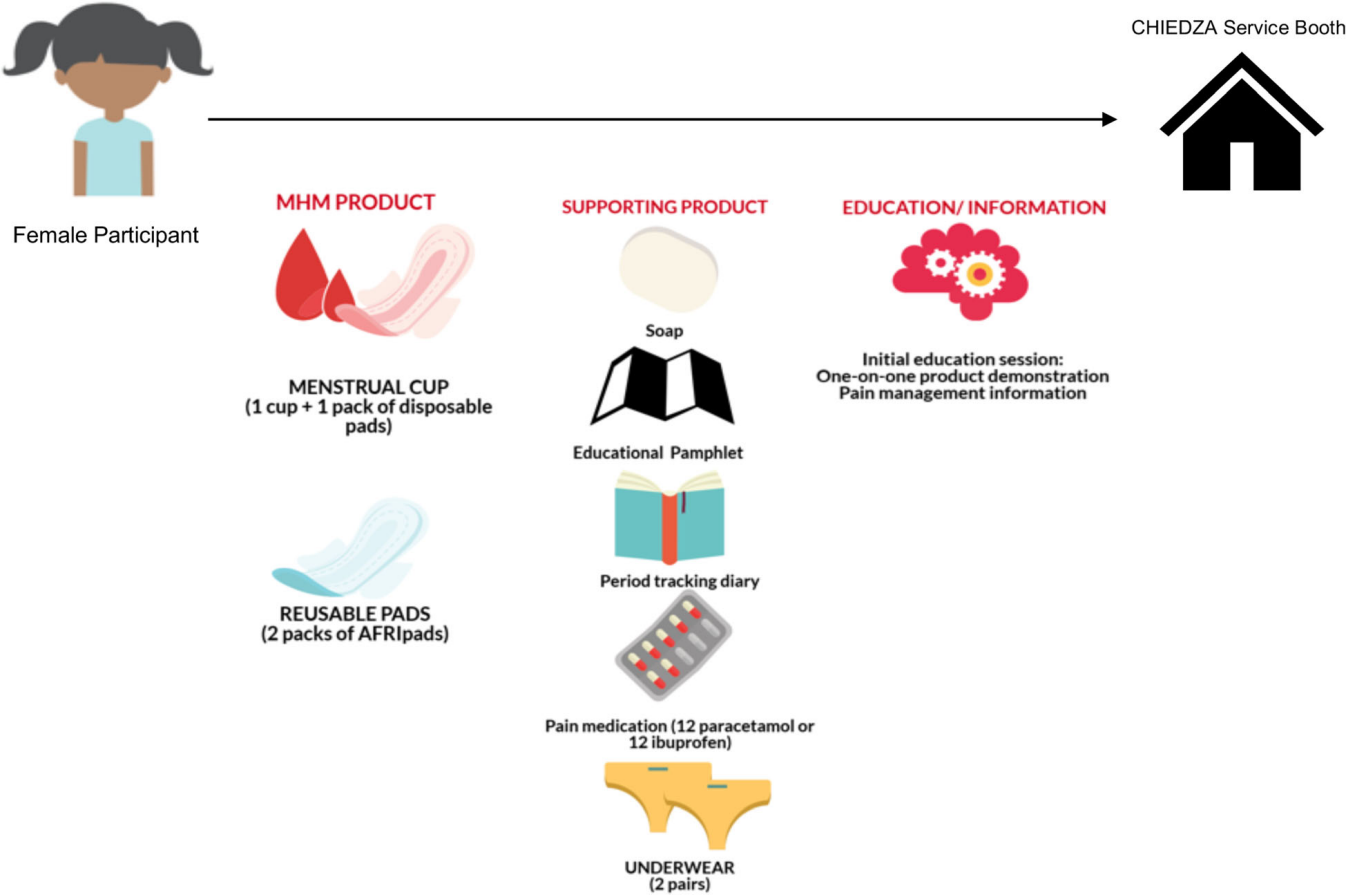
MHH intervention and participant flow during the pilot study

Formative work that included a combination literature review, stakeholder engagement, and qualitative focus group discussions (FGDs), in-depth interviews, and participatory workshops were conducted with young women (aged 16–24 years old), community health workers (CHWs), and other community stakeholders such as relevant community-based organizations and the Ministry of Health and Child Care to inform and develop a Theory of Change (ToC) for the MHH intervention. Details of the formative work will be published elsewhere. The ToC recognizes that successful and sustainable MHH interventions need to address stigma and harmful myths and taboos around menstruation, access to MHH education and products, and access to pain medication. The aim of the ToC was to encapsulate how different components of the intervention would contribute to the intended outcomes (Fig. 2). Based on this, the MHH intervention, to be piloted within the ongoing CHIEDZA trial, was designed to include analgesics (paracetamol or ibuprofen), two pairs of underwear, a bar of soap, a simple period-tracking sheet, comprehensive MHH education including an MHH educational pamphlet, and a choice of either a menstrual cup or reusable pads (Fig. 3). The MHH package was provided free-of-charge and delivered to young women within CHIEDZA trial. The MHH intervention was delivered by an intervention team that included two nurses, three community health workers (CHWs), two youth workers, and one counsellor. The entire intervention team went through a 2-week training that addressed (1) youth-friendly service delivery, (2) logistics management, (3) principles of counselling, (4) research ethics and Good Clinical Practice, and (5) engaging young people and other community members.

### Study procedures

The pilot study was a prospective mixed-methods study conducted from April to July 2019 in all four intervention sites in Harare. In evaluating the implementation of the pilot study, we routinely monitored uptake and coverage data from the CHIEDZA trial and attended weekly CHIEDZA intervention team meetings to more fully understand the context in which the MHH intervention was being conducted [15].

### Quantitative methods

Each attendance at the CHIEDZA service is tracked in real-time using a biometric system [16]. Fingerprints, age, and sex for each participant is recorded and delinked from participant’s name, birthdate, address, or other identifying information. Additionally, the services taken up by participants at each attendance are recorded on electronic tablets. Data for the female participants who accessed CHIEDZA services in the four Harare sites were used to assess intervention uptake and product choice. MHH intervention uptake was calculated as the proportion of women who took up MHH services of those who attended CHIEDZA. *Z* tests were conducted to compare the proportions reporting MHH intervention uptake and MHH product choice disaggregated by age group.

### Qualitative methods

We conducted one focus group discussion (FGD) with the intervention team and two FGDs and four in-depth interviews (IDIs) with female CHIEDZA participants to explore the factors that influenced uptake of the intervention and choice of menstrual product. The intervention team’s FGD included all eight team members: there were three males (aged 24–32 years old) and five females (aged 24–44 years old). The FGD explored their understanding of, and attitude towards, the MHH intervention, their experiences of delivering the intervention, and their own experience of using the available products. For the FGDs with participants, we used purposive sampling to select 12–15 women to participate in two FGDs disaggregated by age (16–19 and 20–24 years old). Participants were selected to include those who did, and did not, take up the MHH intervention, and those who chose the menstrual cup or reusable pads respectively. At midline (two months into the pilot study), participants were screened (over the period of a week) by a female research assistant (RA), informed about the study, and asked if they were willing to be contacted via telephone to participate in the FGDs and subsequent IDIs. Two FGD participants from each age group were purposively selected to represent different product choices (two for the menstrual cup and two for the reusable pads), for follow-up IDIs to explore the factors that informed menstrual product choices.

The FGDs and IDIs were conducted face-to-face by an experienced female RA in either Shona or English (as agreed by the participants), used semi-structured topic guides and were audio-recorded. FGDs took 45–60 min and IDIs took 30–45 min. FGDs took place at the CHIEDZA site outside of usual opening hours to ensure confidentiality. IDIs took place at a time and place most convenient to the participant. Written informed consent was provided before the FGDs or IDIs were initiated.

Audio recordings of the FGDs and IDIs were then directly transcribed into English for analysis. Data was analysed using thematic analysis based on the following broad themes: current MHH knowledge, perceptions, and practices, facilitators and barriers to product choice, and product user experience [17]. Codes were generated based on these themes and sub-themes emerging from the transcripts. All transcripts were coded manually by MT and reviewed by a senior social scientist (JR). Themes and coding were continually reviewed and refined to capture emerging new codes. Verbatim quotes from interview participants were captured to highlight thematic areas and to increase our understanding of the context.

Quantitative and qualitative data were analysed independently and then the findings triangulated to deepen our understanding of how the intervention was working, how it was being received, and how it could be improved. These findings were then further interrogated during the weekly CHIEDZA intervention team meeting. Collectively, the group would reach a consensus on any changes or actions needed based on the evidence from the data and their experiences.

## Results

### MHH intervention uptake

Of the 1732 eligible participants who sought services at the CHIEDZA centres between April and July 2019, 1414 (81.6%) took up the MHH intervention at their first visit. There was no evidence of a difference of uptake between the four sites. There was strong evidence for a difference in uptake by age group with 690/816 (84.6%) of 16–19 year olds compared to 724/916 (79.0%) of 20–24 year olds accessing the MHH package (*p* = 0.003).

From the qualitative data, key themes related to factors that influenced uptake of the MHH intervention were access to free menstrual products and analgesics, youth-friendly intervention delivery, and access to MHH information.

#### Access to free menstrual products and analgesics

Almost all participants, particularly younger women, cited the MHH intervention as the reason behind their initial CHIEDZA visit:

“I personally came here with an intention to get pads and when I entered into the CHIEDZA booth, I saw a very friendly service provider and felt comfortable and free to talk” (FGD, 16–19 years old).

A key motivating factor for the observed high uptake was the provision of free reusable menstrual products, particularly reusable pads. Most participants reported having to use old socks or cotton wool in the absence of the menstrual product they would prefer to use or were able to afford before the economic downturn in Zimbabwe:

“sometimes I would also use cloths when I didn’t have enough pads to last my period” (FGD, 20–24 years old).

Almost all participants reported that they were “*grateful*” and “*happy*” that the intervention provided them with a choice of menstrual products which they could not afford and did not have access to before. MHH intervention team members also reported that the free products were “*the most important hook*” for young women that accessed the CHIEDZA services:

“…those who are coming in for menstrual hygiene, they are going out and inviting others for menstrual hygiene. They are only telling them that if you go to the community you can get pads, you can get a cup or something. They are not really raving about other services but it’s all about menstrual hygiene… the communities love the products we have” (FGD, Nurse).

Some participants reported experiencing pain during their menstrual periods and cited access to monthly analgesics as an additional reason for taking up the MHH intervention.

Almost all participants chose to take up the MHH intervention. Of those that did not, most only declined uptake because their menstrual product of choice was unavailable on the day of their visit.

#### Youth-friendly intervention delivery and access to MHH information

Participants had learned about the MHH intervention in CHIEDZA through community mobilization efforts facilitated by the intervention team:

“…we were on our way from clinic and we were told to go to the community hall to get some pads” (FGD, 20–24 years old).

Once at CHIEDZA, participants reported being “*treated well*” and many found the service providers “*friendly*” and “*helpful*”. MHH intervention uptake was both a function of the provision of needed menstrual products and youth-friendly service provision facilitated by the delivery team. Participants highlighted how the intervention staff, unlike their parents or teachers, provided much needed access to MHH information in a safe, non-judgemental environment and in a way in which resonated with them:

“I think this is a good programme because it helps us. Some children might have questions but they are not able to ask their parent, they might not be open to their parents but CHIEDZA, you are free to ask and say things you want” (FGD, 16–19 years old).

Many participants described how their only conversations about menstruation began and ended at menarche and were limited to menstrual product use and basic hygiene guidance. The MHH intervention provided young women an avenue to learn more about menstrual health from trained staff and to talk through their MHH-related concerns and anxieties in a safe space:

“When I entered into the booth, I saw a very friendly service provider and felt comfortable and free to talk and I was able to express my feelings and to openly seek the help I needed, and I was assisted there. Getting pads was now an extra benefit” (FGD, 16–19 years old).

Participants highlighted the MHH-related education sessions with the intervention staff. These sessions gave participants an opportunity to feel the menstrual products, to observe menstrual product use demonstrations, and to openly discuss myths and taboos around menstruation in private consultation. All the intervention team members reported that the MHH component of CHIEDZA was received with “*gratefulness*” and was the “*most popular*” service.

Overall, both the provision of free menstrual products and youth-friendly service provision were highlighted as key facilitators to intervention uptake. However, older participants seemed to be more motivated by the former rather than the latter as most of them had children with them or household responsibilities to get back to and thus did not have the time to engage in the MHH-related education sessions or other youth-friendly activities within CHIEDZA.

### Menstrual product choice among participants

**Fig. 4:**
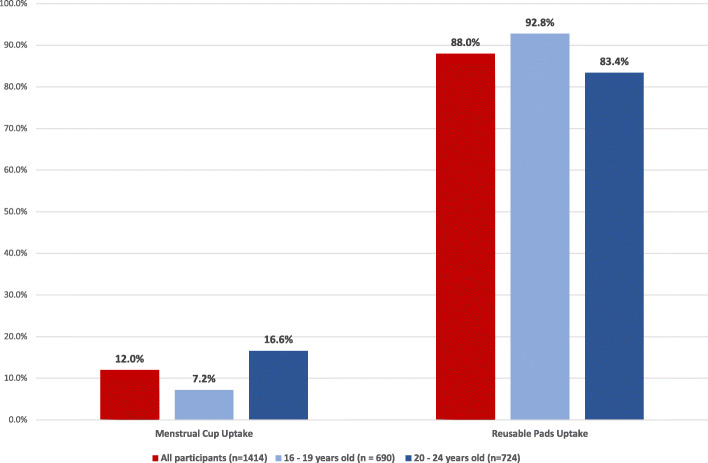
Menstrual product choice disaggregated by age group

Of the 1414 participants who took up the MHH intervention, 1244 (88.0%) participants chose to receive the reusable pads and the remaining 170 (12.0%) chose a menstrual cup on their first visit. There was strong evidence of a difference of product choice by age, with 50/690 (7.2%) of 16–19 year olds to take up the MHH intervention choosing the menstrual cup versus 120/724 (16.6%) of 20–24 year olds (*p* < 0.001) (Fig. 4). The qualitative data with clients and the intervention team highlighted key themes related to factors that influenced product choice**:** barriers and facilitators to the uptake of menstrual cups; and barriers and facilitators to the uptake of reusable pads.

#### Barriers to the uptake of menstrual cups

Sociocultural norms negatively influenced both the delivery and the uptake of the menstrual cup among participants. Participants reported that they and their caregivers in the community were hesitant about inserting the cup into their vagina. Most feared that the cup would “take their virginity”. Some feared that the “big” cup would be too difficult or painful to insert. Others thought the cups were stiff and hard and feared that the cup would stretch out their vagina making them undesirable for men to have sex with. The FGD with the intervention team members also highlighted these fears, and this affected their ability to promote cup uptake, with many explaining their struggles with delivering clear messaging to allay these concerns:

“I think it’s also an issue of needing to look at our cultural values. It’s about what are we told from childhood about virginity and inserting such big things. So that issue is a concern and how would you tackle it if you want to introduce a cup” (FGD, Community health worker).

Participants mentioned that the intervention team appeared reluctant to talk about and distribute the cup, and some expressed that they would have been willing to trying the cup if it had been talked about during service delivery:

“I just heard someone saying cup, but I did not know which cup she was referring to… I got pads instead of cup, and then I was like a cup? I did not get it, I thought she is talking about a cup of tea. I honestly thought that the CHIEDZA people had been unfair, and I wanted to go back and to get a cup” (FGD, 20–24 years old).

#### Facilitators promoting the uptake of menstrual cups

The main factors that facilitated menstrual cup uptake included anecdotal evidence of menstrual pain relief and the prevention of leakage. Some participants chose the cup over the reusable pad as it could be cleaned and dried discreetly. Even though most participants chose the reusable pads, many raised concerns over their need to “keep [their menstruation] a secret”, and challenges in washing and drying their pads outside where other family members and neighbours could see.

The majority of participants gave negative accounts of their menstrual experiences and often described menstrual blood as “dirty” or “impure”. Many spoke about being restricted from housework, social or religious gatherings or sports while others expressed fears of leaking or “spoiling” their clothes leading to being “teased” by boys or chastised by other females during their menstrual periods. Informed by these negative perceptions and experiences of menstruation, some participants chose the menstrual cup over the reusable pads as they felt it was less likely to leak:

“The time for menstruation is so annoying because you will be anxious and you will be afraid of spoiling your clothes, you may not feel comfortable with a pad, so I decided to try a cup” (IDI, 20–24 years old).

Others that chose to take up the menstrual cup also cited “pain relief” as reason for uptake:

“[Other participants] told me that period cup heals period pains slowly. If you continue to use it you end up not having period pains” (FGD, 20–24 years old).

Limited access to water in the communities and seasonal rains also informed product choice. Some of the participants who opted for the cup cited the fact that it did not require much water to wash or time to dry as reusable pads as the reason for their choice:

“the cup is very smart, and you don’t need to do much washing, but you just remove it and empty it” (IDI, 20–24 years old).

#### Barriers and facilitators promoting the uptake of reusable pads

The main facilitators included peer influence (particularly for participant’s aged 16–19 year olds) and the similarity between reusable pads and disposable pads. Participants noted that the reusable pads were most similar to disposable pads they had used before and therefore the less “scary” than the cup. Many of the participants, particularly those aged 16–19 years old, were encouraged to visit CHIEDZA by a classmate that had previously visited the intervention site:

“We were in class and Rashna brought her pads and started to show us, ‘see what they look like, and see what they look like!’ So I said, ‘Where did you get them?' and she said 'I got them from the community centre, on Tuesdays.’ Today is my second time coming here, when I came for the first time, I was given pads” (FGD, 16–19 years old).

Overall, the few barriers to reusable pads uptake seemed to be linked to the environmental factors that facilitated menstrual cup uptake.

## Discussion

In this pilot study of a novel comprehensive MHH intervention, the uptake was high. Key factors to intervention uptake, and the SRH program more broadly, were the availability of free menstrual products and analgesics, youth-friendly intervention delivery, and access to tailored MHH education. Most young women preferred reusable pads to menstrual cups. Barriers to reusable pads uptake were limited to environmental factors such as limited access to the amount of water needed to wash the pads, concerns about appropriately drying pads in the wet season and discomfort around openly drying the pads. Highly influential facilitators to uptake of reusable pads included peer influence amongst younger women, the appearance of the pads, and the comfort and familiarity derived from the similarity of the reusable pads to the more well-known disposable pads. Despite anecdotal evidence of menstrual cups reducing leakage and menstrual pain and being easier to clean than reusable pads, uptake was negatively informed by strong sociocultural beliefs around the preservation of virginity, lack of promotion of the cup from the intervention team, fear around the size of the cup which was perceived to cause vaginal stretching or pain, and/or anxiety around an inability to insert it properly. This study highlights the importance of context-specific interventions and informed choice when offering MHH services to young women.

Our study findings support similar findings from other LMICs that note that most young women face challenges in accessing menstrual products and only learn about menstruation at menarche and even then, the information is limited and skewed by local myths and taboos [18, 19]. These barriers then lead to feelings of isolation, shame, and fear that negatively inform how young women experience menstruation over time [3].

Data on community-based MHH interventions in LMICs is limited [3, 20]. This study provides evidence of the need for MHH interventions in community-based settings. Young women want access to MHH information, analgesics, and menstrual product choice and, if possible, young women positively respond to these services being delivered in youth-friendly spaces by supportive and friendly staff. The principles of Behaviour Centred Design (BCD) theory posit that behaviour change interventions must disrupt the external environment with a “surprise” that causes a shift in the target individual that results in the desired change or “performance” [21, 22]. Nested within a larger SRH intervention, the qualitative data from the pilot study suggests that the MHH component may have been the most attractive service of the SRH package—effectively acting as the “surprise“ that disrupted the community environment causing young women to come to CHIEDZA with the prospect of being rewarded with much needed free menstrual products, information, and support. Our hypothesis is that MHH services can facilitate access to broader SRH and HIV services within CHIEDZA. While the pilot data does not provide quantitative evidence that other SRH services in CHIEDZA were subsequently taken up, we can posit that the MHH intervention increased female engagement with CHIEDZA services. We will be able to investigate this further in the larger study. Robust evidence for the potential “pull factor” of MHH interventions in SRH programming could present a strong case for the integration of MHH in SRH and HIV programming for young women.

Reusable menstrual products are both cost-effective and environmentally friendly. There is also anecdotal evidence from study participants that menstrual cups reduce instances of leakage and period pain, particularly from those participants that previously used tampons to manage their periods. Despite these benefits, barriers to menstrual cup uptake centred around sociocultural norms that discourage insertable products due to (1) fears of “losing virginity” by rupturing the hymen, or (2) fears of hurting or stretching the vagina due to the size of the cup. Young women, particularly unmarried women, are often discouraged from inserting products into their vagina to preserve the hymen as a sign of one’s purity and virginity before marriage [23]. Our results highlight that these sociocultural norms influenced, not only, the participants but also the intervention team members as well. Despite extensive training on menstrual cup use, most of the intervention team members reported to have found it difficult to promote the menstrual cup because of their sociocultural beliefs. The tenets of BCD state that sustainable and effective interventions need to consider factors outside of the behaviour setting as these external factors inform participant behaviour [21]. Our findings suggest that product choice goes beyond provision—external environmental factors such as access to water and sociocultural factors in the community also play roles in the decision-making process. With a growing body of work to scale-up use of the menstrual cup in LMICs, understanding social and contextual factors will prove critical to improving acceptability [24]. Importantly, as reflected in our findings, intervention acceptability has to be considered and addressed from the perspective of both the participants and the service providers [25].

MHH is now recognised as an important public health issue worldwide [26], with an increase in MHH advocacy and research globally [14, 27]. Collaborative networks such as the Menstrual Health Hub and the African Coalition for Menstrual Health Management and advocacy efforts such as the “MHM in Ten” initiative, globally recognized “Menstrual Hygiene Day”, and the annual co-hosting of the MHM in Water, Sanitation and Hygiene (WASH) in Schools virtual conference have all played important roles in placing MHH at the centre of international research and development dialogue and in mobilizing efforts to address the MHH needs of girls and women in LMICs [27–30]. However, apart from a small feasibility study investigating menstrual practices and perceptions around the use of the Duet (an insertable menstrual product) and a cup acceptability study with 54 young women, little scientific data on MHH needs or experiences among young women in Zimbabwe is available [23, 31]. A systematic review of menstrual health interventions conducted in 2020 also highlights limited research into the lived experiences of young women that engage with MHH interventions in LMICs with most of the existing data focusing on school-based interventions and education outcomes [14]. This pilot provides crucial MHH programming information that has contributed to the development and implementation of improved a multi-component MHH intervention that aims to improve MHH knowledge, practices, and perceptions among young women in Zimbabwe.

**Fig. 5:**
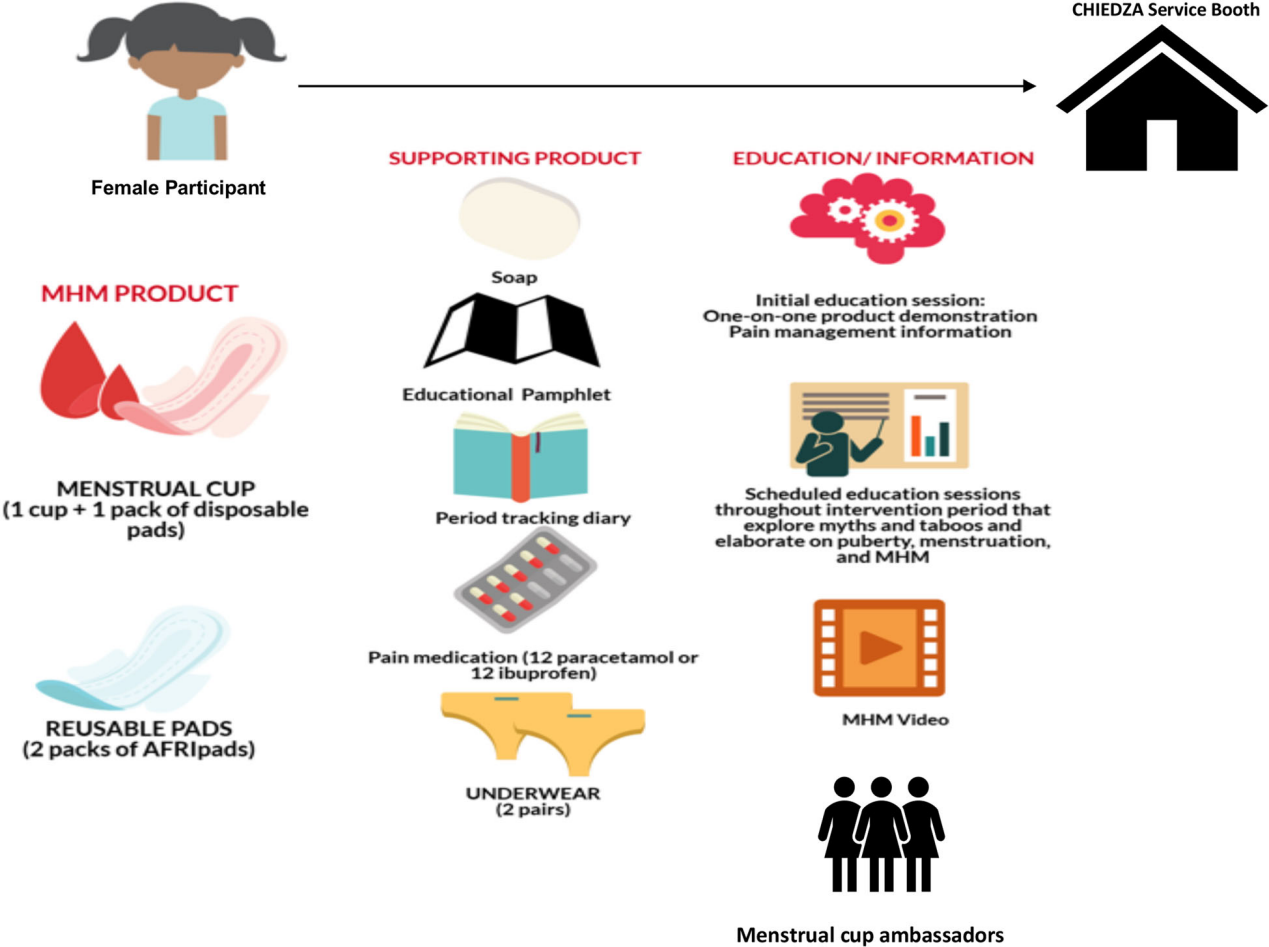
MHH intervention and participant flow for main MHH intervention

Findings from the pilot informed the following changes to the MHH intervention within CHIEDZA (see Fig. 5):

1. Implementation of trained menstrual cup ambassadors to increase cup promotion and sensitization and to provide ongoing support for new cup users
2. Procurement of smaller and softer menstrual cups that are less intimidating to new users and are less likely to cause discomfort or pain during insertion
3. Implementation of group-based MHH education sessions aimed at demystifying menstruation and facilitating MHH dialogue for participants
4. Inclusion of community members such as mothers, fathers, partners, and caregivers, in MHH discussions

Strengths of the study were the availability of real-time quantitative data and the use of a mixed-methods approach to gain an in-depth understanding of the various contextual and individual factors that influenced both the engagement of young women with MHH intervention and menstrual product choice. Limitations of the study are that data were only collected on menstrual product uptake at single point in time and uptake does not necessarily translate to product use. Additionally, pilot data was collected from a fairly small sample in poor urban settings in Harare, Zimbabwe therefore the data may not be generalizable to all young women, such as women in rural or high-income settings. Due to the sensitive nature of the discussions, qualitative data collection may have been informed by social desirability bias leading to inaccurate reporting of factors informing menstrual product choice.

## Conclusions

To our knowledge, this is the first study to investigate MHH intervention uptake and product choice in a community-based setting in an LMIC. Overall, the pilot study results showed an unmet need for comprehensive MHH interventions in the community. Results also highlighted the strong influence of sociocultural and environmental factors on menstrual product choice and economic factors in informing participant engagement overall. Community-based interventions should be context-specific and multicomponent focused to fully address the MHH needs of young women. Importantly, access to MHH education, pain management medication, and a choice of MHH products in youth-friendly SRH programming may act as facilitating factors to increase female engagement in SRH services and improve young women’s SRH outcomes over time.

## Supplementary Information

**Additional file 1.** MHH Intervention Uptake and Product Choice at First Visit. Quantitative data extracted from biometric system highlighting MHH intervention uptake and product choice during pilot period.

## Data Availability

All data generated or analysed during this study are included in this published article (and its supplementary information file).

## Abbreviations

BCD: Behaviour Centred Design
CHW: Community Health Worker
FGD: Focus Group Discussion
IDI: In-Depth Interview
LMIC: Low and Middle Income Countries
MHH: Menstrual Health and Hygiene
MHM: Menstrual Hygiene Management
NGO: Non-Governmental Organisation
RA: Research assistant
SDGs: Sustainable Development Goals
SRH: Sexual and Reproductive Health
UNICEF: United Nations Children's Fund
WASH: Water, Sanitation, and Health
WHO: World Health Organization
